# Haematological changes after tonsillectomy in mild thalassemia children recommendations

**DOI:** 10.1016/j.bjorl.2026.101929

**Published:** 2026-10-14

**Authors:** Jianmin Huang, Shan He, Qiuyun Zhang, Shuwei Ma, Yongjing You

**Affiliations:** a Department of Otorhinolaryngology, Fujian Children's Hospital (Fujian Branch of Shanghai Children’s Medical Center), College of Clinical Medicine for Obstetrics & Gynecology and Pediatrics, Fujian Medical University, Fuzhou, Fujian, China; b Department of Otorhinolaryngology, Shanghai Children's Medical Center, Shanghai Jiao Tong University School of Medicine, Shanghai, China

**Keywords:** Thalassemia, Tonsillectomy, Children, Prospective cohort study, Peripheral blood parameters

## Abstract

**Objective:**

This study aims to evaluate the alterations in peripheral blood parameters and the therapeutic outcomes following tonsillectomy in pediatric patients diagnosed with mild thalassemia.

**Methods:**

We conducted a prospective cohort study involving 65 children who underwent tonsillectomy (with or without adenoidectomy) between May 2023 to March 2025. Patients with thalassemia were assigned to the anaemia group (n = 30), while those without underlying diseases who underwent surgery during the same period were included in the control group (n = 35). The changes in white blood cell count, neutrophil percentage, red blood cell count and hemoglobin level were compared between the two groups at 1-day, 7-days, 1-month, 3-months, and 6-months postoperatively. Postoperative recovery and complications were also compared.

**Results:**

In both groups, white blood cell count, neutrophil percentage, red blood cell count and hemoglobin level showed significant increases at both 1-day and 7-days post-surgery compared to preoperative levels (p < 0.05). The postoperative changes in red blood cell count and hemoglobin level were significantly larger in the anaemia group than the control group at one week and one month after surgery, respectively (p < 0.05). There were no significant differences in the incidence of postoperative snoring relief, bleeding, fever, sore throat, or dizziness between the two groups (p > 0.05).

**Conclusion:**

Tonsillectomy proves to be a safe and effective intervention for alleviating snoring in children with mild thalassemia. After the surgery, regular monitoring of blood parameters and routine transfusions are not required. This conclusion is supported by the absence of significant postoperative haematological deterioration or an increased risk of complications in the mild thalassemia cohort.

**Level of Evidence:**

2b.

## Highlights

• Haematological changes post-tonsillectomy in children with mild thalassemia.
• Thalassemia patients had greater RBC and HB fluctuations after surgery.
• Complication rates were comparable to healthy paediatric controls.
• No transfusions required; surgery did not exacerbate anemia.
• Routine transfusion or monitoring is not recommended perioperatively.

## Introduction

Thalassemia encompasses a range of hereditary haemoglobin disorders that result from the inadequate synthesis of globin peptide chains. This condition can lead to symptoms such as haemolytic anaemia, various developmental disorders, and, in severe cases, premature death. Treatment options for affected individuals often include blood transfusions, splenectomy, or hematopoietic stem cell transplantation.^1^ The prevalence of thalassemia is notably high in regions like Africa, Southeast Asia, and southern China.^2^ Gene screening conducted across 17 regions in southern China reveals a carrier rate of 8.95%. Despite nationwide efforts to promote premarital and prenatal thalassemia gene screening, which have successfully reduced the occurrence of moderate and severe cases, a significant number of children with thalassemia are still born each year.^3^^,^^4^

Obstructive Sleep Apnea (OSA) can lead to serious complications, including airway obstruction, hypoxia, and hindered growth and development in children.^5^ For children with thalassemia, hypoxia can aggravate anemia and hemolysis, further complicating their growth and developmental outcomes.^1^^,^^6^ Consequently, it is critical to provide timely interventions for children exhibiting symptoms such as snoring and enlarged tonsils. Tonsillectomy, often performed with or without adenoidectomy, is a well-established and effective treatment option.^7^^,^^8^ However, there is a lack of research focusing on the postoperative outcomes for these children and whether they require ongoing interventions like blood transfusions.^9^^,^^10^

It remains unclear whether hematological changes after tonsillectomy differ between children with mild thalassemia and healthy controls. Chronic hemolytic anemia and surgical stress may potentially worsen hematological status or increase transfusion needs.^11^ Low hemoglobin levels before surgery are one of the independent risk factors for complications after surgery.^12^ These factors support the need to evaluate routine blood changes after tonsillectomy in thalassemia children, as abnormal responses may indicate higher postoperative risks or the need for targeted interventions. This prospective cohort study investigates hematological dynamics and clinical outcomes to fill current knowledge gaps and provide evidence for perioperative management strategies.

## Methods

### Participants

This prospective observational cohort study enrolled 65-patients who received tonsillectomy (with or without adenoidectomy) between May 2023 and March 2025. Participants were categorized into an anaemia group, or a control group based on the presence of thalassemia. The inclusion criteria were as follows: children with preoperative tonsillar hypertrophy and snoring; those who successfully completed the tonsillectomy (with or without adenoidectomy); patients with comprehensive clinical data and follow-up information; individuals diagnosed with thalassemia through preoperative genetic testing were allocated to the anaemia group, while those with normal preoperative examination results and no underlying conditions were assigned to the control group. Informed consent was obtained from all participating families. The exclusion criteria included: patients with severe cardiopulmonary dysfunction, coagulation disorders, uncontrolled systemic infection and irreversible organ damage; individuals diagnosed with anaemia of an unknown type preoperatively; those who refused to participate; and patients receiving treatment for other conditions during the study period. This study was approved by the Ethics Committee of the Hospital. A sample size power calculation was conducted before the study began to ensure adequate statistical power for detecting differences in primary outcomes between groups. The final sample size was deemed sufficient after accounting for potential loss to follow-up and incomplete data.

### Data collection

Basic patient information, including gender, age, snoring status, surgical type and operative method, were collected one day prior to surgery, along with details about the surgery date and method. During postoperative follow-up, recovery and efficacy were documented within three months, specifically noting snoring relief, postoperative pain, bleeding, fever, and dizziness. Peripheral blood routine results were recorded one day before surgery, and peripheral venous blood samples were obtained for routine testing at one day, seven days, one month, three months, and six months postoperatively. The main observation indicators in blood routine tests included: White Blood Cell (WBC), Neutrophil Percentage (NE%), Red Blood Cell (RBC) count, and Haemoglobin (HB).

### Surgical method

All patients underwent tonsillectomy conducted by the same senior surgeon. During the operation, disposable radiofrequency ablation electrodes were used for separation and hemostasis procedures. Patients diagnosed with adenoid hypertrophy underwent a concurrent adenoidectomy. Throughout the procedure, there were no incidents of major bleeding or emergencies. All patients received prophylactic antibiotics (cefazolin 30 mg/kg; clindamycin for penicillin/cephalosporin allergy). After surgery, all patients received a cold liquid-to-semi-liquid diet, normal saline gargling, and symptomatic therapy for fever and throat pain.

### Outcome assessment indicators

The follow-up process incorporated both outpatient reviews and telephone consultations. The postoperative efficacy indicators included assessments of bleeding, sore throat, fever, and snoring, with a total follow-up duration of three months. The evaluation of bleeding was based on the classification system established by Windfuhr et al.^13^ in 2001: Grade I refers to minimal bleeding that resolves spontaneously; Grade II indicates moderate bleeding that necessitates local compression; Grade III denotes severe bleeding requiring general anaesthesia for suturing; Grade IV describes continuous massive bleeding leading to shock or necessitating external carotid artery haemostasis; and Grade V entails fatal bleeding that cannot be controlled. The pain associated with postoperative sore throat was measured using the Wong-Baker Faces Pain Rating Scale (WB), which ranges from 0- to 10-points, with 1–3 points indicating mild pain, 4–6 points indicating moderate pain, and 7–10 points indicating severe pain. Postoperative snoring and respiratory sounds were classified as worsening, relieved, or absent. Fever was assessed by noting any body temperature exceeding 38.5 °C. Patients and their family members were responsible for recording these observation indicators.

### Statistical analysis

Statistical analysis was conducted using SPSS 26.0 software. Categorical data were compared using the Chi-Square test, continuity-corrected Chi-Square test, or Fisher's exact test, with results presented as n (%). The Shapiro-Wilk test assessed the normality of continuous data. For continuous data that followed a normal distribution, the independent samples *t*-test was employed for comparisons between groups, while repeated measures ANOVA was utilized for comparisons across multiple time points, with Bonferroni correction applied for post-hoc tests, reported as $\bar{\text{x}}\pm \text{s}$. For non-normally distributed continuous data, the nonparametric Mann-Whitney *U*-test was used for between-group comparisons, and the nonparametric Friedman test was applied for multi-time point comparisons, also with Bonferroni correction for post-hoc tests, expressed as M (P25, P75). A p-value of < 0.05 was considered statistically significant.

## Results

### Baseline patient data comparison

The study included 65 paediatric patients, consisting of 30 individuals in the anaemia group (22 with mild α–thalassemia and 8 with mild β-thalassemia; all classified as mild thalassemia with no moderate or severe cases) and 35 in the control group. Patient characteristics are summarized in Table 1. The control group comprised 10 males and 25 females, with ages ranging from 2- to 12-years and a median age of 4.00-years (interquartile range: 4.00–6.00); In the anaemia group, there were 9 males and 21 females, with an age range of 2- to 9-years and a median age of 5.00 (3.00, 7.00) years. There were no significant differences between the two groups regarding gender and age (p > 0.05).

**Table 1:** Comparison of basic data between two groups of patients.

Group	n	Gender		Age
		Male	Female	(years)
Control group	35	10 (28.57)	25 (71.43)	4.00 (4.00,6.00)
Anemia group	30	9 (30.00)	21 (70.00)	5.00 (3.00,7.00)
Z/χ^2^		0.016^a^		−0.588^b^
p		0.900		0.556

Notes: ^a^ Chi-square test, ^b^ Nonparametric Mann-Whitney *U*-test.

Stratified analysis of mild α–versus β-thalassemia within the anaemia group showed no significant differences in HB change trends and RBC change trends at any postoperative time point (p > 0.05). This supports the pooling of α/β-thalassemia data for primary analysis.

## Comparison of postoperative blood test results

### Comparison of postoperative changes in WBC and NE%

As shown in Table 2, compared with preoperative levels, the percentages of WBC and NE% in both the anaemia group and the control group increased at 1-day and 7-days postoperatively (p < 0.05), but there was no significant difference at 1-month and later postoperatively (p > 0.05). There were no significant differences between the two groups in terms of changes at each postoperative time point relative to the preoperative levels (p > 0.05).

**Table 2:** Comparison of WBC and NE% after surgery between two groups.

Indicators	Time Points	Values		Changes from baseline			
		Control group	Anaemia group	Control group	Anaemia group	*t*/Z	p
		(n = 35)	(n = 30)	(n = 35)	(n = 30)		
WBC (10^9^/L)	Pre-op	7.61 (6.13, 8.87)	7.65 (6.68, 8.10)				
	POD1	9.66 (7.77, 12.56)*	10.11 (8.98, 13.30)*	2.56 (0.98, 4.85)	2.58 (1.23, 5.64)	−0.697	0.486
	POD7	8.71 (7.33, 12.80)*	9.24 (7.64, 10.63)*	1.21 (0.18, 4.88)	1.60 (0.36, 2.65)	−0.138	0.890
	POD1M	7.61 (6.52, 8.68)	7.82 (6.51, 8.48)	0.03 ± 1.81	0.17 ± 1.55	−0.324	0.747
	POD3M	7.15 (6.47, 8.17)	7.15 (6.33, 7.88)	0.10 (−1.50, 0.72)	−0.07 (−0.85, 0.27)	−0.566	0.571
	POD6M	7.33 (6.34, 7.92)	7.26 (6.54, 7.81)	−0.09 (−1.06, 0.64)	−0.12 (−0.67, 0.13)	−0.52	0.603
	χ^2^	57.091	70.521				
	p	<0.001	<0.001				
NE (%)	Pre-op	48.70 (42.10, 56.40)	42.80 (39.15, 50.95)				
	POD1	60.10 (51.90, 69.90)*	60.38 (45.35, 70.00)*	12.80 (3.20, 18.30)	9.50 (4.30, 20.18)	−0.263	0.792
	POD7	55.10 (50.30, 68.30)*	52.50 (44.73, 67.35)*	6.50 (−0.50, 15.20)	5.45 (0.00, 15.23)	−0.072	0.942
	POD1M	54.70 (48.90, 60.80)	46.20 (40.90, 52.65)	3.85 ± 11.47	2.49 ± 12.16	0.463	0.645
	POD3M	49.00 (44.10, 57.30)	47.85 (43.33, 52.75)	0.77 ± 9.37	1.97 ± 7.49	−0.566	0.573
	POD6M	49.10 (43.40, 52.70)	47.30 (41.78, 50.88)	−0.44 ± 6.49	1.01 ± 7.46	−0.838	0.405
	χ^2^	35.822	37.138				
	p	<0.001	<0.001				

Note: The normal range for WBC count is 4.4–11.9 (10^9^/L); the normal range for NE% is 31.00–65.00 (%); Pre-op, One day before surgery; POD, Postoperative day; PODM, Postoperative Month.

### Comparison of postoperative changes in RBC

Data shown in Table 3 revealed that the control group exhibited a significantly increased RBC count one day after surgery (p < 0.05). However, no significant changes were noted at 7-days postoperatively or thereafter (p > 0.05). In contrast, the anaemia group demonstrated a significant increase in RBC count both 1-day and 7-days post-surgery (p < 0.05), but showed no significant alterations at one month and beyond (p > 0.05). When comparing the two groups, the anaemia group showed significant differences in RBC count at one day, one month, three months, and six months postoperatively (p < 0.05). No significant differences in RBC count were observed between the two groups at 7-days post-surgery (p > 0.05).

**Table 3:** Comparison of RBC counts between two groups (10^9^/L).

Time Points	RBC		Changes from baseline			
	Control group	Anaemia group	Control group	Anaemia group	*t*/Z	p
	(n = 35)	(n = 30)	(n = 35)	(n = 30)		
Pre-op	4.62 ± 0.29	5.15 ± 0.59				
POD1	4.82 ± 0.30^a^	5.56 ± 0.66^a^	0.20 ± 0.28	0.41 ± 0.32	−2.781	0.007
POD7	4.71 ± 0.35	5.42 ± 0.65^a^	0.10 ± 0.39	0.27 ± 0.34	−1.846	0.070
POD1M	4.64 ± 0.34	5.34 ± 0.58	−0.05 (−0.22, 0.20)	0.14 (−0.02, 0.44)	−2.204	0.027
POD3M	4.56 ± 0.34	5.25 ± 0.56	−0.05 ± 0.24	0.10 ± 0.34	−2.178	0.033
POD6M	4.54 ± 0.30	5.23 ± 0.61	−0.08 ± 0.24	0.08 ± 0.33	−2.316	0.024
*F*	8.954	13.594				
p	0.005	<0.001				

Note: ^a^ Indicates p < 0.05 compared with preoperative values within the group, and the same applies hereafter. Normal range: 4.00～5.50 (109/L); Pre-op, One day before surgery; POD, Postoperative Day; PODM, Postoperative Month.

### Comparison of postoperative changes in HB

Table 4 illustrates that HB levels in the control group significantly increased one day after surgery (p < 0.05), with no statistically significant changes detected at 7-days and later (p > 0.05). The anaemia group also showed a significant increase in HB levels at both one day and 7-days post-surgery (p < 0.05), but no significant changes were noted at 1-month and thereafter (p > 0.05). When comparing the two groups, the increase in HB levels in the anaemia group at one-month post-surgery was significantly higher than pre-surgery levels (p < 0.05). However, there were no significant differences in HB changes between the two groups 1-day, 7-days, 3-months, and 6-months post-surgery compared to pre-surgery levels (p > 0.05).

**Table 4:** Comparison of HB levels between two groups (g/L).

Time Points	HB		Changes from baseline			
	Control group	Anaemia group	Control group	Anaemia group	*t*/Z	p
	(n = 35)	(n = 30)	(n = 35)	(n = 30)		
Pre-op	126.00 (123.00, 132.00)	106.00 (100.75, 112.00)				
POD1	134.00 (129.00, 137.00)*	116.00 (110.75, 119.50)*	6.23 ± 7.27	9.00 ± 5.53	−1.707	0.093
POD7	130.00 (126.00, 135.00)	113.00 (107.00, 117.00)*	3.60 ± 10.22	6.40 ± 6.40	−1.343	0.185
POD1M	126.00 (123.00, 133.00)	110.50 (105.50, 115.25)	0.00 (−6.00, 6.00)	3.00 (−0.25, 6.50)	−2.248	0.025
POD3M	126.00 (121.00, 130.00)	108.00 (103.00, 114.00)	−0.54 ± 5.87	1.47 ± 5.08	−1.463	0.148
POD6M	126.00 (122.00, 131.00)	106.00 (101.00, 113.00)	−0.86 ± 4.97	0.73 ± 6.01	−1.168	0.247
χ^2^	39.347	68.291				
p	<0.001	<0.001				

Note: Normal range for HB test: 112～149 (g/L); Pre-op, one day before surgery; POD, postoperative day; PODM, postoperative month.

### Comparison of postoperative recovery

The incidence of postoperative complications, including bleeding, fever, sore throat, and dizziness, did not differ significantly between the two groups (p > 0.05). Specifically, six children from both groups experienced bleeding, all classified as grade I. Both groups reported sore throat symptoms within the first week after surgery, primarily mild pain, with a few cases escalating to moderate or severe discomfort. Pain relief was effectively achieved through oral administration of acetaminophen or ibuprofen, and sore throat symptoms resolved in both groups within one-month post-surgery. Detailed information is shown in Table 5.

**Table 5:** Comparison of postoperative adverse reactions between two groups n (%).

Adverse reaction	Group	n	POD1	POD7	POD1M	POD3M
Bleeding	Control group	35	1 (2.86)	3 (8.57)	0 (0.00)	0 (0.00)
	Anaemia group	30	0 (0.00)	2 (6.67)	0 (0.00)	0 (0.00)
	χ^2^		^d^	0.000^c^		
	p		1.000	1.000	1.000	1.000
Fever	Control group	35	4 (11.43)	6 (17.14)	0 (0.00)	0 (0.00)
	Anaemia group	30	3 (10.00)	5 (16.67)	0 (0.00)	0 (0.00)
	χ^2^		0.000^c^	0.003^a^	‒	‒
	p		1.000	0.959	1.000	1.000
Sore throat (moderate to severe)	Control group	35	8 (22.86)	12 (34.29)	0 (0.00)	0 (0.00)
	Anaemia group	30	7 (23.33)	11 (36.67)	0 (0.00)	0 (0.00)
	χ^2^		0.002^a^	0.040^a^	‒	‒
	p		0.964	0.841	1.000	1.000
Dizziness	Control group	35	0 (0.00)	0 (0.00)	0 (0.00)	0 (0.00)
	Anaemia group	30	1 (3.33)	0 (0.00)	0 (0.00)	0 (0.00)
	χ^2^		^d^	‒	‒	‒
	p		0.462	1.000	1.000	1.000

Note: ^a^ Was analysed using Chi-Square test, ^c^ Was analysed using continuity-corrected chi-square test, and ^d^ Was analysed using Fisher's exact test; Pre-op, One day before surgery; POD, Postoperative Day; PODM, Postoperative Month.

As shown in Table 6, both groups experienced a notable reduction in snoring following surgery, but there was no statistically significant difference between the two groups.

**Table 6:** Comparison of snoring conditions between two groups after surgery n (%).

Group	n	POD1			POD7			POD1M			POD3M	
		No change	Relieved	Worsening	No change	Relieved	Disappeared	No change	Relieved	Disappeared	No change	Disappeared
Control group	35	2 (5.71)	33 (94.29)	0 (0.00)	2 (5.71)	31 (88.57)	2 (5.71)	2 (5.71)	0 (0.00)	33 (94.29)	2 (5.71)	33 (94.29)
Anaemia group	30	0 (0.00)	28 (93.33)	2 (6.67)	0 (0.00)	30 (100.00)	0 (0.00)	0 (0.00)	1 (3.33)	29 (96.67)	0 (0.00)	30 (100.00)
χ^2^		3.293d			2.905d			2.450d			d	
p		0.168			0.245			0.345			0.495	

Note: Pre-op, one day before surgery; POD, postoperative day; PODM, postoperative month.

## Discussion

Thalassemia results from gene mutations or deletions that impair haemoglobin production, leading to ineffective erythropoiesis, reduced red blood cell lifespan, and chronic anaemia. Mild cases can be assessed through standard blood tests, while severe cases may require treatments like blood transfusions or hematopoietic stem cell transplantation.^1^ Families are deeply concerned about the impact of surgery on thalassemia and whether special interventions are necessary after the procedure. However, research in this area is limited. This study utilized a prospective approach to conduct routine postoperative blood tests on children with mild thalassemia, enabling the observation of changes in inflammatory markers such as WBC and NE%, as well as indicators of anemia like HB and RBC levels. The findings from this study will inform the development of postoperative follow-up treatment plans and provide families with a clearer understanding of surgical risks and expected recovery, ultimately fostering their understanding and cooperation.

In patients with thalassemia, RBCs show changes in deformability and increased instability, making them more susceptible to damage and haemolysis.^14^^,^^15^ This study indicates that in the anaemia group, RBC counts increased on both the 1st-day and 7th-day post-surgery. When comparing the anaemia group to the control group, the fluctuations in RBC counts were significantly more pronounced in the control group at 1-day, 1-month, 3-months, and 6-months post-surgery. These findings suggest that children with thalassemia are more vulnerable to the effects of surgical stimulation, resulting in marked variations in RBC counts compared to the general population.

Multiple studies have established a correlation between low HB levels and increased rates of infection, heightened disease severity, and poorer prognoses.^11^^,^^12^^,^^16^ For children with non-transfusion-dependent thalassemia, RBC transfusions are necessary when HB levels drop rapidly due to infection, surgery, or haemolytic crises, aiming to maintain HB levels at or above 90 g/L.1 In this study, the preoperative anemia group maintained HB levels of at least 87 g/L, and HB levels increased on both the first and seventh days after surgery across both groups. Notably, there was no significant decrease in HB levels postoperatively, which means no blood transfusions were required. The postoperative HB levels remained stable and even showed a slight increase in the short term, likely due to postoperative volume reduction leading to a relative concentration and elevation of haemoglobin levels.

In our study, both groups of children exhibited elevated percentages of WBC and NE% at 1- and 7-days post-surgery, yet they experienced low rates of postoperative fever and bleeding. The postoperative inflammation observed in the oropharynx was relatively mild, likely due to the antibacterial properties of lysozyme, immunoglobulins and other components found in saliva, coupled with local hygiene practices such as mouth rinsing.^17^ Infection can trigger inflammatory responses that may inhibit HB production. Therefore, while monitoring wound hygiene is critical for preventing infection, routine CBC monitoring is not necessary for children with mild thalassemia post-tonsillectomy, as haematological parameters return to baseline without intervention and do not correlate with complication risk.

In our study, both groups experienced significant relief from snoring following surgery, resulting in satisfactory surgical outcomes. Valentim-Coelho et al. reported that after 6-months of positive airway pressure therapy for OSA patients, RBC and HB levels decreased.^18^ Conversely, Elverland et al. found that HB levels increased 6-months after tonsillectomy and adenoidectomy.^19^ In our research, RBC and HB levels in both groups showed a slight increase within 7-days post-surgery, returning to preoperative levels after one month. The discrepancies in findings among studies may stem from variations in patient demographics and treatment methods. Our study specifically targeted paediatric patients with airway obstruction due to mild thalassemia (non-iron deficiency anaemia). Despite these differences, all studies suggest that improving airway obstruction can positively influence RBC and HB levels.

Postoperative bleeding and sore throat are prevalent complications following tonsillectomy.^20^ This study detected no significant differences in the incidence of these complications between the two groups, and the overall bleeding rate was less than 10%. These findings suggested that mild anaemia was not a risk factor for postoperative bleeding or sore throat. This aligns with findings by Bal^21^ and Xu et al.^22^ However, conducting preoperative blood tests may still help mitigate the risk of bleeding. Both groups of children reported experiencing sore throat within the first week post-surgery, though only a few cases were of moderate severity, and the severity levels were comparable. Non-Steroidal Anti-Inflammatory Drugs (NSAIDs) were effective in alleviating pain, reducing inflammatory responses associated with pain, and promoting wound healing.^23^ Current literature indicates no significant relationship between the risk of post-tonsillectomy bleeding and the use of NSAIDs.^24^ Moreover, children's perceptions of pain and their ability to tolerate it after surgery are influenced by various factors, including age, psychological state, personality, and environment. Involving parents in postoperative pain management can contribute to pain relief and enhance the overall quality of life after surgery.^25^

This study has several limitations, including its experimental design (such as long-term vital sign data at scheduled follow-up time points were not collected) and focus solely on mild thalassemia. During the study period, clinical surgical interventions primarily targeted patients with mild thalassemia, and there is no data on surgical treatment for children with moderate to severe thalassemia, which is a critical future research direction. Additionally, the postoperative follow-up data were limited to 6-months after surgery, lacking long-term follow-up.

## Conclusions

Our findings suggest that tonsillectomy is a safe and effective treatment for snoring in children with mild thalassemia. Routine blood transfusions and blood test monitoring are not required after surgery. The study's data shows no significant postoperative haematological deterioration, no increased complication risk, and a return of peripheral blood parameters to baseline within 1-month post-surgery in the mild thalassemia cohort. Preoperative blood testing for baseline anaemia assessment is recommended, but no modified perioperative haematological management is needed.

Future research should evaluate tonsillectomy outcomes in children with moderate and severe thalassemia to determine whether targeted perioperative interventions (e.g., prophylactic transfusions, intensive monitoring) are necessary and include long-term follow-up to assess the sustained effects of airway obstruction relief on haematological parameters in thalassemia children.

## ORCID ID

Shan He: 0000-0003-0589-7235

Qiuyun Zhang: 0000-0002-1973-6638

Shuwei Ma: 0009-0007-5001-9964

## Authors’ contributions

Jianmin Huang and Shan He carried out the studies, participated in collecting data, and drafted the manuscript. Jianmin Huang, Qiuyun Zhang, Yongjing You and Shuwei Ma performed the statistical analysis and participated in its design. All authors read and approved the final manuscript.

## Ethics committee statement

The ethical approval of this study was obtained from the Ethics Committee of our institution. The original approval document is written in Chinese. Due to the official document management regulations of the institution, we are unable to provide an English version of the approval document at present. This study was performed in strict accordance with the ethical principles for medical research involving human subjects. All ethical requirements have been fully met.

## Ethical approval and informed consent statements

This study was approved by the Ethics Committee of Fujian Children's Hospital (nº 2023ETKLR05105). Written informed consent was obtained from all participants’ legal representatives.

## Funding

This study was supported by the Startup Fund for scientific research, Fujian Medical University (Grant nº 2022QH1214).

## Data availability

The authors declare that all data are available in repository.

## Declaration of competing interest

The authors declare no conflicts of interest.
